# Dissipation and Residue of Metalaxyl-M and Azoxystrobin in Scallions and Cumulative Risk Assessment of Dietary Exposure to Hepatotoxicity

**DOI:** 10.3390/molecules27185822

**Published:** 2022-09-08

**Authors:** Yida Chai, Rong Liu, Xiaoying Du, Longfei Yuan

**Affiliations:** 1 School of Agriculture, Yangtze University, Jingzhou 434025, China; 2 State Key Laboratory of Integrated Management of Pest Insects and Rodents, Institute of Zoology, Chinese Academy of Sciences, Beijing 100101, China; 3 Institute of Crop Sciences, Chinese Academy of Agricultural Sciences, Beijing 100081, China

**Keywords:** cumulative risk assessment, probabilistic model, metalaxyl-M, azoxystrobin, residue

## Abstract

Metalaxyl-M and azoxystrobin have been used to control various fungal diseases on scallion and other crops. In view of the adverse toxic effects of both on the mammalian liver, it is necessary to conduct a cumulative risk assessment of their dietary exposure to consumers. The residues of metalaxyl-M and azoxystrobin on scallion were determined by a quick, easy, cheap, effective, rugged, and safe method (QuEChERS) combined with high-performance liquid chromatography tandem mass spectrometry (LC-MS/MS). The half-lives were about 1.15 and 3.89 days, respectively, and the final residues after a seven-day harvest interval were <0.001–0.088 mg/kg and 0.190–4.687 mg/kg, respectively. The cumulative dietary risk quotient of the two fungicides to Chinese consumers calculated by the probability model is 13.94%~41.25%. According to the results of the contribution analysis, the risk posed by azoxystrobin is much greater than that of metalaxyl-M. Although metalaxyl-M and azoxystrobin do not pose a cumulative risk to Chinese consumers, the risk to children and adolescents is significantly higher than that to adults. This suggests that in future research, more consideration should be given to the cumulative risk of compounds to vulnerable groups.

## 1. Introduction

As a common spice, scallion is widely eaten in many parts of Asia and some Latin American countries. Scallion contains a variety of active ingredients, has a broad-spectrum antibacterial effect, and is very valuable for the development of medicine and food [1]. In addition, many studies have proved that scallion contains active ingredients to inhibit cancer and treat cardiovascular diseases [2,3,4]. However, with the continuous expansion of scallion production and planting areas, soil pathogenic bacteria have increased, and the occurrence and damage of scallion diseases continues to increase [5]. Rational use of chemical pesticides is needed to reduce losses. Metalaxyl-M and azoxystrobin are used as systemic fungicides to control *Peronosporales* of scallion.

Metalaxyl [methyl *N*-(2,6-dimethylphenyl)-*N*-(methoxyacetyl)-D-alaninate] is a phenylamide broad-spectrum fungicide which inhibits the incorporation of uridine into RNA through the interaction with RNA polymerase-1, thus it is widely used across the world to control various crops disease caused by *Peronosporales* [6,7,8]. Metalaxyl is a racemate; the product has been gradually replaced by metalaxyl-M (Figure S1A) which is composed of main bioactive enantiomeric (*R*-enantiomer) component and is therefore also called high-efficiency metalaxyl [9]. The original intention of replacing metalaxyl with metalaxyl-M was to reduce the amount of this pesticide application by almost half and to reduce the risk of residues [10]. The 2002 JMPR (The Joint FAO/WHO Meeting on Pesticide Residues) report stated that toxicological studies at the time found no toxicological differences between metalaxyl and metalaxyl-M, and that the dose–response relationship was similar [11]. However, up until now, many studies have shown that metalaxyl-M may cause more serious toxicological effects on environmental organisms and mammals than racemic metalaxyl. For instance, Zhang et al. found that metalaxyl-M induced more severe inflammation, necrosis and vacuolization in mouse liver cells than racemic metalaxyl [12]. The studies by OuYang et al. [13] and Liu et al. [14] also indicate that metalaxyl-M is significantly toxic to aquatic (*Tubifex tubifex*) and terrestrial (*Eisenia fetida*) non-target animals. It is necessary to pay attention to the new risks caused by the large application of metalaxyl-M.

Azoxystrobin (Figure S1B), methyl (*E*)-2-[[6-(2-cyanophenoxy)-4-pyrimidinyl]oxy]-α-(methoxymethylene)benzeneacetate, is one of the new methoxy acrylate fungicides developed with natural product strobilurins as the lead compound [15]. The main mechanism of action is to inhibit mitochondrial respiration by blocking the electron transfer between cytochrome b and cytochrome c_1_, inhibiting both mycelial growth and spore germination [16,17]. As a typical broad-spectrum systemic fungicide, azoxystrobin has a good control effect on most diseases caused by fungi such as Ascomycetes, Basidiomycetes, Deuteromycetes and Oomycetes, and it is precisely because it can control many types of fungal diseases at the same time that azoxystrobin has achieved great success and is widely used around the world [18,19]. The JMPR report shows that the acute oral LD_50_ (lethal dose) in rats is >5000 mg/kg bw, and the acute dermal LD_50_ is > 2000 mg/kg bw, and so falls within low-toxicity pesticides. However, the toxicological evaluation also points out that azoxystrobin has certain liver toxicity to mammals [20].

Since pesticides pose potential hazards to public health, and dietary intake is the most direct and major source of population exposure to pesticides, it is necessary to monitor and evaluate pesticide residues in food in order to protect consumers [21,22]. Due to the large registered crop species and application rates of metalaxyl-M and azoxystrobin, consumers are often exposed to both due to multiple residues on the same crop and/or residues on multiple different crops, as shown by the monitoring results of pesticide residues in various places [23,24]. In the JMPR report, the toxicological descriptions of these two low-toxicity pesticides mentions their adverse effects on the mammalian liver, and that the main target organ is the liver; both can lead to different degrees of increase in mammalian liver weight [11,20]. Therefore, when assessing the potential health hazards of these two pesticide residues in food, it may be more meaningful to take into account the additive toxic effects of the two pesticides, and to assess their cumulative exposure risk.

The US Environmental Protection Agency (EPA), the European Union Food Safety Authority (EFSA), and the World Health Organization WHO/IPCS have respectively summarized the currently available methods for assessing the cumulative risk of mixtures [25,26,27,28,29], including the hazard index (HI), point of departure index (PODI), margin of exposure (MOE), and the relative potency factor (RPF) method, etc. Each method has its own calculation method to determine the toxicity of the dose–additive effects due to exposure, but in general the RPF method is more applicable and is the preferred method [30]. Cumulative exposure concentration obtained by these methods are compared with health reference values, such as the acceptable daily intake (ADI), the acute reference dose (ARfD), the no observed adverse effect level (NOAEL) or benchmark dose limit (BMDL) to obtain the cumulative risk.

In the present study, QuEChERS method combined with LC-MS/MS was established to determine the residues of metalaxyl-M and azoxystrobin on scallion in four regions of China. Combined with residue data from other crops, a probabilistic model was used to fit the cumulative dietary exposure of the two pesticides and to assess chronic dietary risks to consumers.

## 2. Materials and Methods

### 2.1. Reagents and Standards

Metalaxyl-M standard (purity 99.3%) and azoxystrobin standard (purity 98.7%) were purchased from Dr. Ehrenstorfer GmbH (Augsburg, Germany). Chromatographic purity acetonitrile was purchased from Merck KGaA. Analytical-grade sodium chloride (NaCl) and anhydrous magnesium sulfate (MgSO_4_) were obtained from Sinopharm Chemical Reagent Co., Ltd. (Shanghai, China) and Beijing Tongguang Fine Chemical Co. (Beijing, China), respectively. Chromatographic purity formic acid was purchased from CNW Technologies (Shanghai, China). The purification fillers of octadecylsilane (C18), primary secondary amine (PSA), and graphitized carbon black (GCB) were purchased from Agela Technologies (Tianjin, China), and a syringe filter (nylon, 0.22 µm) was purchased from ANPEL Laboratory Technologies Inc. (Shanghai, China). The 39% metalaxyl-M·azoxystrobin suspension emulsion (SE) used in the field trial tests was provided by Syngenta Investment Co., Ltd. (Shanghai, China).

To obtain a 1000 mg/L standard solution, 10 mg metalaxyl-M standard and azoxystrobin standard was accurately weighed and dissolved in acetonitrile, which was then maintained at 4 °C in the dark. A 2.5 mL amount of the standard solution was accurately weighed and diluted with acetonitrile. An appropriate amount of the standard mixed solution was accurately transferred and diluted with blank scallion matrix extract to prepare 0.0005, 0.001, 0.005, 0.01, 0.05, 0.1, 0.5, 0.75, and 1.0 mg/L matrix-matched standard solutions for sample quantification.

### 2.2. Extraction and Purification Process of the Samples

Firstly, 10 g (±0.01 g) homogenized scallion samples were weighed into a 50 mL centrifuge tube (the quality control and additive recovery samples were allowed to stand for 15 min after adding the standard solution). A 20 mL amount of chromatographic acetonitrile was added and shaken for 5 min in a high-throughput tissue grinder. After ultrasonic extraction for 15 min, 5 g of sodium chloride was added and then shaken by hand for 2 min. This was centrifuged at 6000 rpm for 5 min, and then the supernatant was taken and purified with a modified QuEChERS tube. After high-speed centrifugation at 10,000 rpm for 5 min, the supernatant was filtered through a 0.22 μm membrane and put into the injection bottle for LC-MS/MS analysis.

The effects of 12 different purification adsorbents on the response results were compared, including 50 mg PSA, 50 mg C18, 50 mg GCB, 25 mg PSA with 150 mg MgSO_4_, 50 mg PSA with 150 mg MgSO_4_, 25 mg C18 with 150 mg MgSO_4_, 50 mg C18 with 150 mg MgSO_4_, 25 mg GCB with 150 MgSO_4_, 50 mg GCB with 150 mg MgSO_4_, 50 mg PSA + 50 mg C18 with 150 mg MgSO_4_, 50 mg PSA + 50 mg C18 + 50 mg GCB with 150 mg MgSO_4_, and blank. The optimal adsorbent was selected by comparing the addition recovery at the level of 0.002 mg/kg.

### 2.3. Instrumentation

Metalaxyl-M and azoxystrobin were analyzed by high-performance liquid chromatography (Agilent 1290 Infinity II Multisampler, Agilent Technologies, City of Santa Clara, CA, USA) and a tandem triple quadrupole mass spectrometer (6470 Triple Quadrupole LC/MS System, Agilent Technologies, City of Santa Clara, CA, USA) with an electrospray ionization (ESI) source operated in positive ion mode (ESI+). An Agilent EclipsePlusC18 RRHD column (50 mm × 2.1 mm, 1.8 μm) was used for chromatographic separation at a temperature of 40 °C. The mobile phases were acetonitrile (A) and 0.1% (*v/v*) formic acid aqueous solution (B). The gradient elution procedure is shown in Table S1. The flow was 0.30 mL/min, and the sample injection volume was 1.5 μL. The gas temperature was set at 280 °C and the gas flow rate at 7 L/min for MS detection working conditions. The sheath gas temperature was set at 320 °C and the sheath gas flow was 11 L/min, and the capillary voltages were controlled at 4000 V under positive ion detection mode. Analytes were determined in multiple reaction monitoring (MRM) mode. For instrument control, data acquisition, and processing, MassHunter Workstation version 10.1 (Agilent Technologies, City of Santa Clara, CA, USA) was used. Under the operating conditions given above, the two compounds were quantified based on the acquisition parameters as listed in Table S2.

### 2.4. Method Validation

The analytical method was validated from the aspects of linearity, sensitivity, matrix effect, precision and stability [31].

The matrix effect can be expressed by the equation as follows [32]:

Matrix effect (ME, %) = (Slope of calibration curves in matrix–slope of calibration curves in solvent)/slope of calibration curves in solvent (× 100%).

The different concentrations of the standard solution were prepared with blank matrix extract to eliminate the excessive matrix effect. The prepared standard solutions with different concentration gradients (0.0005, 0.001, 0.005, 0.01, 0.05, 0.1, 0.5, 0.75, and 1.0 mg/L) were analyzed under the above analysis conditions, and the standard curves were drawn according to the ratio of the absolute number of compounds to the peak areas to verify linearity. The accuracy and precision of the method was verified by five repeated recovery experiments at four different spiked concentrations (0.001, 0.01, 1, and 7 mg/kg).

### 2.5. Field Trial Tests

The open-field trials were designed in accordance with the Guideline on Pesticide Residue Trials (NY/T 788-2018) published by the Ministry of Agriculture and Rural Affairs, P. R. China. Field trials were implemented in four different scallion producing areas in China, include Changping, Beijing (116.41 E, 40.22 N, continental monsoon climate), Taian, Shandong (116.97 E, 36.10 N, temperate monsoon climate), Changsha, Hunan (113.31 E, 28.31 N, subtropical monsoon climate), and Guiyang, Guizhou (106.63 E, 26.32 N, subtropical humid and mild climate). The 39% metalaxyl-M·azoxystrobin SE (10.6% metalaxyl-M and 28.4% azoxystrobin) was sprayed at 351 g a.i./ha active ingredient, and the application was carried out three times with an interval of seven days. The treatment and control samples were sampled multiple times at different time intervals (including 0, 5, 7, 10, and 14 d) after the last application. At least 12 normally growing and healthy scallions with a total weight at least 2 kg were collected and each test for degradation was conducted in triplicate, and for final residue in duplicate. The blank control treatment was the same as the experimental treatment, and the protective line was established between each plot. All scallion samples were cut into sections after removal of soil and roots and preserved at −20 °C until use.

### 2.6. Cumulative Exposure Estimation

Toxicological data in the FAO JMPR assessment report for these two pesticides were selected to ensure data reliability and consistency. The NOAEL for liver toxicity of metalaxyl-M and azoxystrobin were 8 mg/kg bw per day (13-weeks study in dogs) and 18.2 mg/kg bw per day (2-year study in rats), respectively. Even though metalaxyl-M is more toxic than azoxystrobin, azoxystrobin was chosen as the index compound in order to minimize uncertainty due to the fact that the latter has more residue data and its residues are much higher than the former in many crops. The comparison of the NOAEL of the compound with the index compound gives its RPF relative to the index compound.

The cumulative residue of metalaxyl-M and azoxystrobin can be calculated with the following Equation (1) [33]:

(1) $$C_{index,j}=\underset{i=1}{\sum }\left(C_{i,j}\times RPF_{i}\right)$$

where the $C_{index,j}$ is the cumulative concentration expressed as the index compound in food j, $C_{i,j}$ is the concentration of component compound i in food j, and $RPF_{i}$ is the relative potency factor of component compound *i* relative to the index compound.

The cumulative exposure of metalaxyl-M and azoxystrobin can be calculated with the following Equation (2) [33]:

(2) $$E=\underset{j=1}{\sum }E_{j}=\underset{j=1}{\sum }\frac{CONS_{j}\times C_{index,j}}{bw}$$

where the E is the cumulative exposure of the population, $E_{j}$ is the cumulative exposure of the population in individual food j, $CONS_{j}$ is the food j consumption of the population, and bw is the body weight of the population.

### 2.7. Cumulative Dietary Risk Assessment on Probabilistic Method

Monte Carlo simulation was used to fit the input parameters such as pesticide residues, food consumption and body weight, so as to obtain the cumulative exposure closer to the real situation. Inspired by the probabilistic evaluation method recommended by EFSA [34], we divided the entire evaluation process into inner and outer loops. The inner loop obtains the distribution of population chronic dietary exposure risk variability by performing 2000 random Monte Carlo iterations of the input parameters. The outer loop executes the inner loop multiple times (such as 500 times) to obtain the uncertainty distribution of the iteration evaluation results. Monte Carlo sampling was completed in the @risk version 7.6.2 (Palisade, Ithaca, NY, USA) software.

The risk characterization of metalaxyl-M and azoxystrobin can be carried out using the following Equation (3) [33]:

(3) $$R=\frac{E}{NOAEL_{index}}\times UF\times 100\%$$

where R is chronic dietary risk of the population, E is the cumulative exposure of the population and comes from Equation (2), $NOAEL_{index}$ is the no observed adverse effect level of the index compound, UF is the uncertainty factor. The uncertainty factor is divided into two parts, which reflect the uncertainty between species and the uncertainty within species, respectively. For metalaxyl-M and azoxystrobin, both uncertainty factors are taken as 10, that is, the total uncertainty factor is taken as 100.

## 3. Results and Discussion

### 3.1. Optimization of the Analytical Method

**Figure 1:**
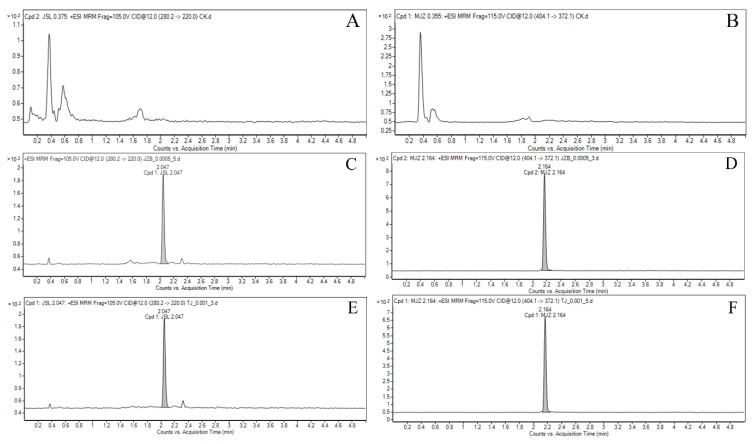
Typical LC-MS/MS chromatograms of metalaxyl-M and azoxystrobin in scallion samples: (**A**,**B**) blank sample, (**C**,**D**) matrix standard at 0.0005 mg/L, and (**E**,**F**) spiked sample at 0.001 mg/kg.

The existence of [M + H] + was determined by the target ion scanning method, and the detection monitoring (MRM) mode of the target compounds was optimized by multiple reactions. The chromatograms of metalaxyl-M and azoxystrobin are shown in Figure 1.

Various adsorbents including GCB, C18 and PSA are widely used to remove different impurities in the matrix, mainly for pigment removal, sugar and fatty acid removal and reverse phase extraction, respectively [35,36]. The effects of 12 different purification adsorbents on the response results were compared by 0.002 mg/kg recovery experiment and the results are shown in Figure S2.

Except for 50 mg GCB and 50 mg GCB with 150 mg MgSO_4_, the recoveries of other adsorbent combinations including blank are mostly between 80–100%, with little difference. In contrast, the absorbent 50 mg PSA + 50 mg C18 with 150 mg MgSO_4_ provided the best recovery results of all adsorbent combinations, and the recoveries of metalaxyl-M and azoxystrobin at the level of 0.002 mg/kg reached 97.79% ± 2.28% and 94.34% ± 7.79% respectively. Therefore, it was selected as the adsorbent combination for subsequent treatment in this study.

### 3.2. Method Validation

The regression equations of the solvent standard curves of metalaxyl-M and azoxystrobin were $y=175194x+4646.8,\;R^{2}=0.9919$ and $y=505999x+9186.5,\;R^{2}=0.9946$, respectively. The regression equations of the matrix standard curves of metalaxyl-M and azoxystrobin in scallions were $y=71002x+149.65,\;R^{2}=0.9996$ and $y=237639x+1759.7,\;R^{2}=0.9983$, respectively. The ME of these two pesticides on scallions were as high as −59.47% and −53.04%, respectively. Therefore, it is necessary to use the blank scallion matrix extract to prepare the standard solution in order to eliminate the matrix effect.

As shown in Table S3, the average recovery of metalaxyl-M in scallion was 97.66–106.27% at spiked levels of 0.001, 0.01, 1, and 7 mg/kg with RSD of 2.11–6.88%, and the average recovery of azoxystrobin was 88.82–105.19% with RSD of 5.30–12.64%. The results indicate that the accuracy and precision of this method meet the requirements of pesticide residue analysis.

The limit of quantification (LOQ) was defined as the minimum spiked concentrations of target analytes in the matrix [37,38]. According to the recovery experiments, under the above analysis conditions, the LOQ of metalaxyl-M and azoxystrobin in scallion was 0.001 mg/kg. The above validation results suggest that this analytical method is reliable for the determination of the two fungicide residues in scallion.

### 3.3. Dissipation Behavior

**Figure 2:**
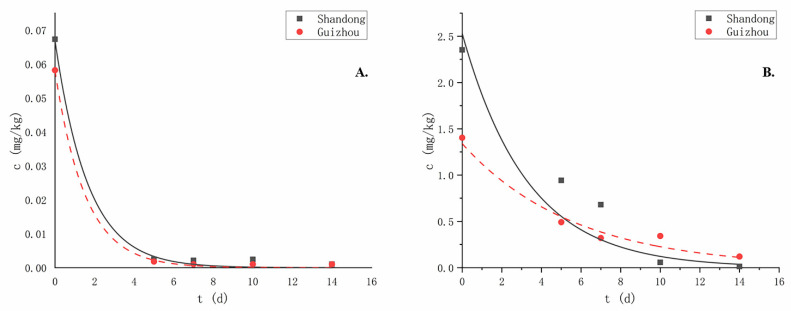
Degradation rates of metalaxyl-M and azoxystrobin in scallion samples in Shandong and Guizhou: (**A**) metalaxyl-M, and (**B**) azoxystrobin.

Research about pesticide degradation is an important part of the risk assessment. Almost all pesticides degrade over time after application and their residues decrease, and these two fungicides are no exception (Figure 2); the dissipation process was of first-order reaction kinetics:

(4) $$C_{t}=C_{0}\times e^{-kt}$$

(5) $$t_{1/2}=\ln2⁄k$$

where $C_{t}$ (mg/kg) is the concentration of the pesticide residue at time t (days), $C_{0}$ (mg/kg) is the initial concentration, k is the degradation rate constant (day^−1^), and $t_{1/2}$ (days) is the degradation half-life of the pesticide which was determined from the k value.

The initial deposition of metalaxyl-M in Shandong and Guizhou were in the range of 0.055–0.080 mg/kg and 0.049–0.067 mg/kg, respectively; seven days after the last application, the degradation rate reached 96.88–98.28%, and the half-lives were 1.15 and 1.06 days, respectively, which are similar to or lower than other research results [39]. The initial deposition of azoxystrobin in these two places were in the range of 2.115–2.590 mg/kg and 1.284–1.523 mg/kg, respectively; seven days after the last application, the degradation rate reached 71.09–77.20%, and the half-lives were 2.28 and 3.89 days, which are similar to other research results [40,41]. The different dissipation rates and initial deposition may be related to the crop species, climate types and planting modes at different sites.

### 3.4. Final Residue Testing

As shown in Table 1, the final residues of metalaxyl-M and azoxystrobin in scallion were determined by the above analysis method. The harvest interval was 7 and 10 days; the residue of metalaxyl-M in scallion at harvest was <0.001−0.088 and <0.001−0.049 mg/kg, respectively. The residue of azoxystrobin at harvest was 0.190−4.687 mg/kg and 0.043−4.368 mg/kg, respectively. Therefore, at the pre-harvest interval (PHI) of seven days, the final residues of azoxystrobin in scallion were lower than the China maximum residue limit (MRL) 7 mg/kg, the EU MRL 10 mg/kg for Welsh onion [42] and the US MRL 7.5 mg/kg for Onion, green, subgroup 3-07B [43]. China has not yet formulated the MRL of metalaxyl-M on scallions, but the final residues were lower than the EU MRL 0.3 mg/kg for Welsh onion [44] and the US MRL 10 mg/kg for Onion, green, subgroup 3-07B [45]. These data will provide reference for metalaxyl-M·azoxystrobin SE reasonable application in scallion and the formulation of MRL of metalaxyl-M on scallion in China.

### 3.5. Cumulative Dietary Risk Assessment

According to the differences between urban and rural areas, gender and age, Chinese consumers were divided into a total of 40 groups. The different body weight and food consumption data were obtained from the Report on Nutrition and Health Status of Chinese Residents (2002). Based on the residue results obtained in this study and on other pesticide residue monitoring trials in China, dietary exposures for different food categories were calculated according to the relative potency factor method. In accordance with the principle of maximizing risk, it is assumed that consumers are exposed to the food with the largest residue of each category. We classify scallions into the soy sauce food category in the risk assessment work, according to the suggestions. All residue data and food categories are shown in Table 2.

The cumulative dietary risk assessment results are shown in Tables S4 and S5. All results did not exceed 100%, including the maximum in the most extreme cases (upper 95% confidence interval of P99.9 for rural females aged 2–3 years, 90.68%). Considering the variability of residual and consumption data, the P97.5 level was selected as an appropriate assessment of chronic risk. Considering the uncertainty of iteration, the upper limit of 95% confidence interval was chosen to be an appropriate level to achieve adequate protection. Based on these results, the risk faced by Chinese consumers is 13.94–41.25%. Among these, the minimum value of 13.94% appeared in the urban male group aged 30–44, while the maximum value of 41.25% was the risk result faced by rural girls aged 2–3. At the same time, the principle of risk maximization was followed in this study as much as possible. For example, the data of the food with the largest residue in a food category was extrapolated to the whole category, which brings a certain uncertainty and makes the evaluation results more conservative. Therefore, metalaxyl-M and azoxystrobin residues do not pose a risk of cumulative exposure hazard to the liver of Chinese consumers.

**Figure 3:**
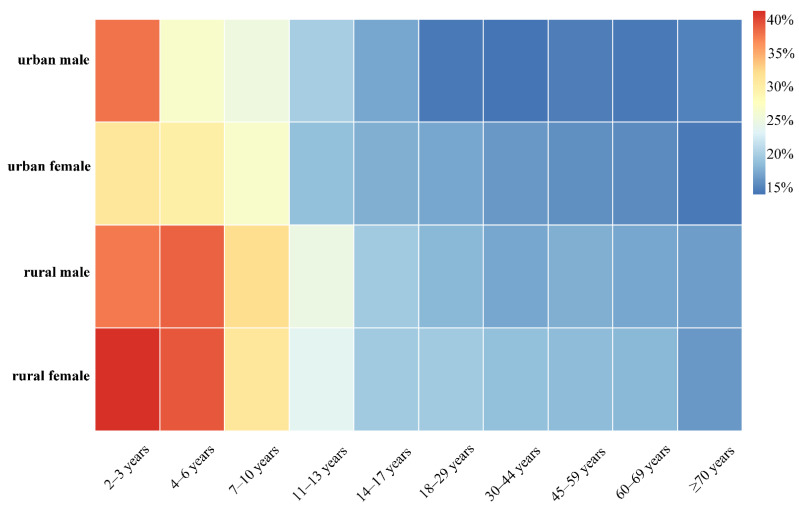
Heatmap of cumulative dietary exposure risk outcomes for different consumer groups (upper 95% confidence interval at P97.5 level).

In order to compare the possible differences between the risks faced by different populations, all the risk results are presented as a heat map, as shown in Figure 3. Intuitively, age led to a large difference in risk outcomes. Children and adolescents are at significantly greater risk than adults. The most important reason is that differences in body weight led to differences in tolerance to toxicity without adequately reducing compound exposure, resulting in a nearly three-fold difference in risk outcomes. The average weight of all groups in rural areas is smaller than that of urban residents of the corresponding gender and age, and the dietary structure of residents also differs between urban and rural areas; as a result, the level of risk faced by rural residents is slightly higher than that of urban residents.

In order to compare the differences in the contribution of different pesticides and food categories to dietary exposure risk, while avoiding the possible effects of age, region and gender, contribution analysis was carried out for 2–3 years old infants, 30–44 years old adults, and 60–69 years old elderly. The analysis results are shown in Table S6. As previously stated, azoxystrobin contributed 76.22–84.58% of the total dietary risk, which was much higher than that of metalaxyl-M despite the latter being more toxic, due to the greater residues of azoxystrobin. For food categories, dark vegetables, light vegetables and fruits are the three main sources of risk, contributing 26.64–43.05%, 24.15–34.85%, and 7.81–32.95%, respectively. In addition to this, rice and products and soy sauce contributed 5.81–8.63% and 3.29–5.35%, respectively. The contribution of different food categories varies greatly: on the one hand, due to the huge difference in intake in the dietary structure, and on the other hand, due to the residues in different crops tending to differ.

**Figure 4:**
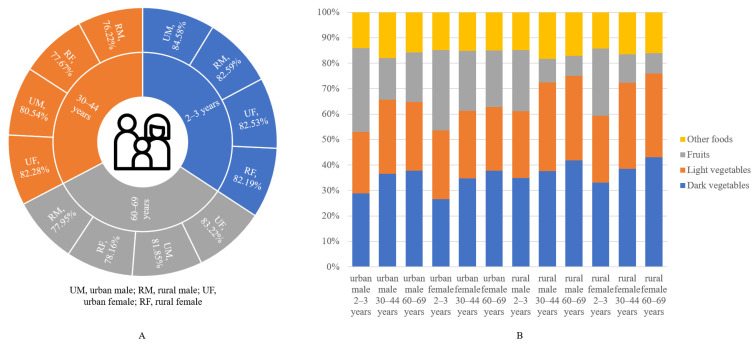
Contribution to dietary exposure of different consumer groups of (**A**) metalaxyl-M and azoxystrobin and (**B**) major food categories.

Different populations have different contribution distributions due to variety in dietary structure. By comparing the contribution of azoxystrobin in Figure 4A, it can be seen that the distribution of contributions of different pesticides across all populations is not significantly different. Mainly due to insufficient data in the assessment to distinguish pesticide residues in foods ingested by different populations, the differences in pesticide contributions are mainly affected only by different food intakes. The same is true for different food categories. Figure 4B summarizes the contribution distribution of the three main risk source food categories for different populations. Taking fruits as an example, the contribution distribution shows that children are at higher risk than adults and the elderly, urban areas are higher than rural areas, and men are slightly lower than women, which is consistent with the trend of the proportion of fruits in the dietary structure of the population. In summary, there is little difference in the contribution distribution of risks faced by different groups of people, mainly because even if there are differences in dietary structure, the residual concentration of each food category does not change, so it is not enough to have a significant impact on the contribution results.

These results of cumulative dietary risk assessment suggest that, in future research, there should be still room for improvement in current dietary exposure assessments, focusing on expanding the amount of consumption and residue data, reducing uncertainty in the assessment, and incorporating more variables. For example, the foods acceptable to consumers in different regions often come from different origins, so their residues are not the same, and the consumption of food categories is often different due to differences in ethnic and regional cultures. Another example is the possible impact of changes in consumption and residues caused by factors such as seasons on the risk assessment results of subchronic and acute exposure to compounds. The subdivision of food categories is another way to reduce the uncertainty of the assessment. In short, expanding the amount of data and incorporating more considerations can effectively reduce the uncertainty and ensure that the evaluation results have sufficient accuracy.

## 4. Conclusions

The residues of metalaxyl-M and azoxystrobin on scallion were determined by QuEChERS method combined with LC-MS/MS. The half-lives were about 1.15 and 3.89 days, respectively, and the final residues after a seven-day harvest interval were <0.001–0.088 mg/kg and 0.190–4.687 mg/kg, respectively. The cumulative exposure risk of the two pesticides to the liver was assessed by the RPF method and probabilistic model, and azoxystrobin was chosen as the index compound. The results of cumulative dietary risk quotient are 13.94%~41.25%; rural females aged 2–3 years face the greatest exposure risk. Although less toxic, azoxystrobin contributed about 80% of the dietary exposure risk, mainly due to its higher residue levels in multiple foods. From the results, it can be seen that while the cumulative dietary exposure to the hepatotoxicity of both metalaxyl-M and azoxystrobin did not exceed the threshold risk to Chinese consumers, vulnerable groups such as children and adolescents faced significantly higher risks than the adult population. The main reason is that although the dietary structure of different age groups differs according to the current statistics, it does not differ enough to make up for the difference in tolerance to toxicity caused by different body weights. Therefore, in the assessment of dietary exposure risk, it is necessary to divide consumer groups, especially for the risks faced by vulnerable groups.

## Figures and Tables

**Table 1:** Final residues of metalaxyl-M and azoxystrobin in scallion samples.

Location	Harvest Interval (d)	Final Residue (mg/kg)			
		Metalaxyl-M ^a,b^		Azoxystrobin ^c,d^	
		1	2	1	2
Beijing	7	0.003	0.003	0.363	0.190
	10	0.005	0.004	0.377	0.492
Shandong	7	0.002	0.002	1.420	1.360
	10	0.003	0.002	0.071	0.043
Guizhou	7	<0.001	<0.001	0.314	0.326
	10	<0.001	<0.001	0.350	0.334
Hunan	7	0.082	0.088	4.687	4.687
	10	0.049	0.049	4.368	4.203

^a^ Supervised trial median residue (STMR) of 7 d and 10 d were 0.0025 and 0.0035 mg/kg, respectively. ^b^ Highest residue (HR) of 7 d and 10 d were 0.088 and 0.049 mg/kg, respectively. ^c^ Supervised trial median residue (STMR) of 7 d and 10 d were 0.861 and 0.364 mg/kg, respectively. ^d^ Highest residue (HR) of 7 d and 10 d were 4.687 and 4.368 mg/kg, respectively.

**Table 2:** Food categories and residue data screened for cumulative risk assessment.

Food Category	Pesticide	Crop	STMR (mg/kg)	HR (mg/kg)	$C_{index}{\;}^{a,b}$ (mg/kg)
Rice and products	Metalaxyl-M	-	-	-	0.160
	Azoxystrobin	Rice	0.160	0.230	
Flour and products	Metalaxyl-M	-	-	-	0.023
	Azoxystrobin	Wheat	0.023	0.180	
Other cereals	Metalaxyl-M	Corn	0.020	0.020	0.051
	Azoxystrobin	Corn	0.005	0.005	
Potatoes and products	Metalaxyl-M	Potato	0.011	0.039	0.045
	Azoxystrobin	Potato	0.020	0.020	
Legumes and products	Metalaxyl-M	Soybean	0.020	0.020	0.246
	Azoxystrobin	Soybean	0.200	0.265	
Dark vegetables	Metalaxyl-M	Tomato	0.034	0.051	0.996
	Azoxystrobin	Water spinach	0.920	4.000	
Light vegetables	Metalaxyl-M	Cauliflower	0.109	0.454	0.488
	Azoxystrobin	Loofah	0.240	0.540	
Fruits	Metalaxyl-M	Watermelon	0.010	0.010	0.723
	Azoxystrobin	Grape	0.700	3.220	
Vegetable oil	Metalaxyl-M	-	-	-	0.050
	Azoxystrobin	Peanut	0.050	0.160	
Soy sauce	Metalaxyl-M	Scallion	0.0025	0.088	0.867
	Azoxystrobin	Scallion	0.861	4.687	

^a^$C_{index}$ was the cumulative concentration expressed as the index compound. ^b^ The index compound was azoxystrobin; The RPF of metalaxyl-M was 2.275.

## Data Availability

Data are contained within the article.

## Supplementary Materials

The following supporting information can be downloaded at: https://www.mdpi.com/article/10.3390/molecules27185822/s1, Figure S1: Structures of metalaxyl-M and azoxystrobin: (A) metalaxyl-M, (B) azoxystrobin. Table S1: Gradient elution procedure. Table S2: Experimental parameters and chromatographic conditions of metalaxyl-M and azoxystrobin. Figure S2: Recovery of different modified QuEChERS purification conditions for targets in scallion matrix at the level of 0.002 mg/kg (n = 5): (a) 50 mg primary secondary amine (PSA), (b) 50 mg octadecylsilane (C18), (c) 50 mg graphitized carbon black (GCB), (d) 25 mg PSA with 150 mg MgSO_4_, (e) 50 mg PSA with 150 mg MgSO_4_, (f) 25 mg C18 with 150 mg MgSO_4_, (g) 50 mg C18 with 150 mg MgSO_4_, (h) 25 mg GCB with 150 MgSO_4_, (i) 50 mg GCB with 150 mg MgSO_4_, (j) 50 mg PSA and 50 mg C18 with 150 mg MgSO_4_, (k) 50 mg PSA and 50 mg C18 and 50 mg GCB with 150 mg MgSO_4_, and (l) blank. Table S3: Recoveries of metalaxyl-M and azoxystrobin in scallions (n = 5). Table S4: Cumulative dietary risk assessment of metalaxyl-M and azoxystrobin for male. Table S5: Cumulative dietary risk assessment of metalaxyl-M and azoxystrobin for female. Table S6: Contribution distribution of dietary risks faced by different population groups.

## Sample Availability

Samples of the compounds are not available from the authors.
