# Supplementary material for: Dissipation and Residue of Metalaxyl-M and Azoxystrobin in Scallions and Cumulative Risk Assessment of Dietary Exposure to Hepatotoxicity
Source: Molecules. 2022 Sep 8;27(18):5822. doi: 10.3390/molecules27185822 (PMC9506456; doi:10.3390/molecules27185822)
Supplement: Supplementary file 1 [file molecules-27-05822-s001.zip › molecules-1896919-supplementary.pdf]

Electronic Supporting Information for

Dissipation and Residue of Metalaxyl-M and Azoxystrobin in Scallions and Cumulative Risk Assessment of Dietary Exposure to Hepatotoxicity

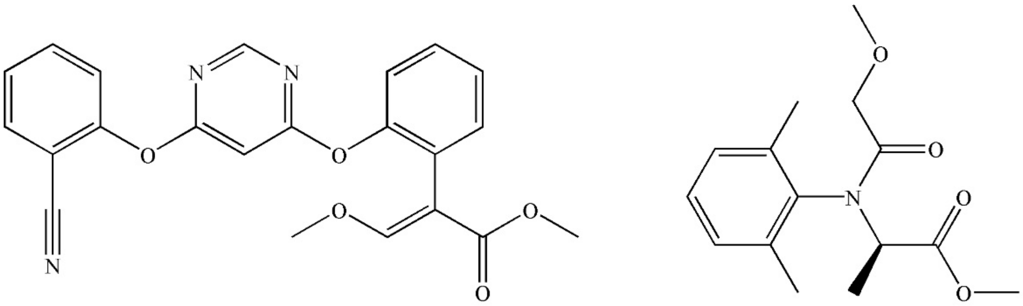

A B

Figure S1. Structures of metalaxyl-M and azoxystrobin: (A) metalaxyl-M, (B) azoxystrobin .

Table S1. Gradient elution procedure.

t/min	Flow rate (mL/min)	Acetonitrile (%)	0.1% Formic acid aqueous solution (%)
0	0.30	10.0	90.0
0.5	0.30	10.0	90.0
1.5	0.30	90.0	10.0
3.5	0.30	90.0	10.0
3.6	0.30	10.0	90.0
5.0	0.30	10.0	90.0

Table S2. Experimental parameters and chromatographic conditions of metalaxyl-M and azoxystrobin.

Compound	Molecular formula	Precursor ion (m/z)	RT (min)	Ion source	Fragmentor Voltage (V)	Quantification ion transition (m/z)	CE1 (eV)	Qualification ion transition (m/z)	CE2 (eV)
Metalaxyl-M	$C_{15}H_{21}NO_4$	280.2	2.047	ESI+	105	280.2/220.0	12	280.2/192.0	18
Azoxystrobin	$C_{22}H_{17}N_3O_5$	404.1	2.157	ESI+	115	404.1/372.1	12	404.1/344.1	26

RT, retention time; CE, collision energy.

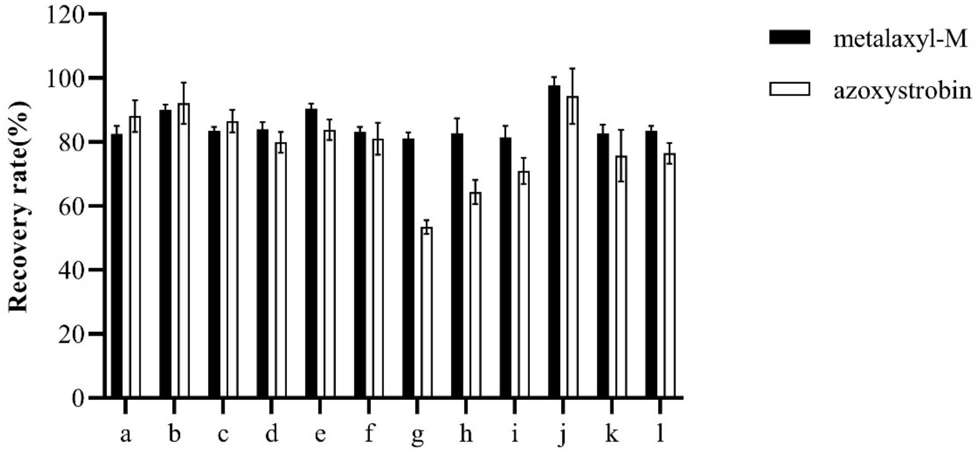

Figure S2. Recovery of different modified QuEChERS purification conditions for tar-gets in scallion matrix at the level of 0.002 mg/kg (n=5): (a) 50 mg primary secondary amine (PSA), (b) 50 mg octadecylsilane (C18), (c) 50 mg graphitized carbon black (GCB), (d) 25 mg PSA with 150 mg MgSO4, (e) 50 mg PSA with 150 mg MgSO4, (f) 25 mg C18 with 150 mg MgSO4, (g) 50 mg C18 with 150 mg MgSO4, (h) 25 mg GCB with 150 MgSO4, (i) 50 mg GCB with 150 mg MgSO4, (j) 50 mg PSA + 50 mg C18 with 150 mg MgSO4, (k) 50 mg PSA + 50 mg C18 + 50 mg GCB with 150 mg MgSO4, and (l) blank.

Table S3. Recoveries of metalaxyl-M and azoxystrobin in scallions (n = 5).

Compound	Spiked level (mg/kg)	Average recoveries (%)	RSD (%)
Metalaxyl-M	0.001	103.68	3.95
	0.01	97.66	2.11
	1	101.53	4.89
	7	106.27	6.88
Azoxystrobin	0.001	101.38	12.64
	0.01	88.82	10.49
	1	100.15	6.51
	7	105.19	5.30

Table S4. Cumulative dietary risk assessment of metalaxyl-M and azoxystrobin for male.

	Percen-tiles	Urban					Rural				
		Lower Limit of Confidence Interval		Upper Limit of Confidence Interval		Mean	Lower Limit of Confidence Interval		Upper Limit of Confidence Interval		Mean
		50%	95%	50%	95%		50%	95%	50%	95%	
2-3 years	P90	23.62%	22.99%	24.34%	24.99%	23.98%	23.59%	22.92%	24.24%	24.92%	23.90%
	P97.5	34.49%	33.13%	36.06%	37.82%	35.34%	34.50%	32.96%	35.99%	37.68%	35.27%
	P99.9	60.12%	54.33%	70.23%	86.33%	66.23%	60.84%	53.44%	69.87%	83.29%	65.68%
4-6 years	P90	16.73%	16.29%	17.22%	17.70%	16.97%	23.98%	23.33%	24.70%	25.46%	24.34%
	P97.5	24.47%	23.33%	25.56%	26.67%	25.00%	35.23%	33.54%	36.85%	38.60%	36.01%
	P99.9	42.63%	37.85%	49.42%	58.36%	46.29%	61.40%	54.73%	70.96%	84.54%	66.75%
7-10 years	P90	15.66%	15.26%	16.18%	16.62%	15.92%	19.78%	19.26%	20.36%	21.04%	20.08%
	P97.5	22.90%	21.90%	24.03%	25.04%	23.47%	29.02%	27.76%	30.41%	32.04%	29.73%
	P99.9	40.04%	35.77%	46.54%	54.85%	43.57%	51.15%	45.55%	59.81%	70.77%	55.98%
11-13 years	P90	12.31%	11.99%	12.69%	13.07%	12.50%	15.32%	14.94%	15.82%	16.30%	15.58%
	P97.5	17.95%	17.21%	18.85%	19.74%	18.39%	22.57%	21.67%	23.57%	24.68%	23.08%
	P99.9	31.39%	27.66%	35.70%	42.83%	33.84%	39.78%	35.57%	46.03%	54.27%	43.20%
14-17 years	P90	10.68%	10.42%	10.97%	11.27%	10.83%	12.16%	11.81%	12.57%	12.91%	12.36%
	P97.5	15.57%	14.98%	16.30%	17.06%	15.94%	17.88%	17.16%	18.80%	19.53%	18.35%
	P99.9	27.30%	24.29%	31.17%	36.66%	29.46%	31.70%	28.84%	36.62%	41.75%	34.26%

18-29 years	P90	9.05%	8.79%	9.32%	9.56%	9.18%	11.37%	11.06%	11.72%	12.02%	11.55%
	P97.5	13.21%	12.67%	13.93%	14.44%	13.57%	16.71%	16.02%	17.54%	18.27%	17.11%
	P99.9	23.13%	20.68%	26.60%	31.64%	25.02%	29.49%	25.98%	33.80%	40.87%	31.94%
30-44 years	P90	8.64%	8.38%	8.89%	9.15%	8.76%	10.56%	10.27%	10.88%	11.21%	10.72%
	P97.5	12.61%	12.13%	13.27%	13.94%	12.95%	15.55%	14.91%	16.30%	17.10%	15.94%
	P99.9	22.20%	19.80%	25.60%	30.44%	24.06%	27.51%	24.57%	31.95%	37.46%	30.02%
45-59 years	P90	9.03%	8.74%	9.29%	9.53%	9.15%	10.78%	10.47%	11.15%	11.51%	10.97%
	P97.5	13.25%	12.68%	13.81%	14.58%	13.55%	15.94%	15.26%	16.80%	17.74%	16.39%
	P99.9	23.20%	21.10%	26.91%	32.26%	25.33%	28.68%	25.27%	33.55%	39.91%	31.24%
60-69 years	P90	8.99%	8.76%	9.24%	9.50%	9.12%	10.66%	10.37%	10.94%	11.24%	10.79%
	P97.5	13.19%	12.66%	13.81%	14.39%	13.50%	15.72%	15.16%	16.46%	17.17%	16.10%
	P99.9	23.12%	20.75%	26.69%	32.28%	25.17%	27.89%	24.84%	32.01%	38.44%	30.24%
≥70 years	P90	9.20%	8.97%	9.47%	9.75%	9.34%	10.07%	9.78%	10.36%	10.69%	10.22%
	P97.5	13.52%	12.93%	14.14%	15.02%	13.84%	14.93%	14.33%	15.62%	16.44%	15.29%
	P99.9	23.92%	21.26%	27.83%	33.19%	25.98%	26.72%	23.94%	31.40%	37.08%	29.24%

Table S5. Cumulative dietary risk assessment of metalaxyl-M and azoxystrobin for female.

Age	Percen-tiles	Urban					Rural				
		Lower Limit of Confidence Interval		Upper Limit of Confidence Interval		Mean	Lower Limit of Confidence Interval		Upper Limit of Confidence Interval		Mean
		50%	95%	50%	95%		50%	95%	50%	95%	
2-3 years	P90	19.35%	18.75%	19.90%	20.68%	19.64%	25.74%	25.13%	26.53%	27.35%	26.16%
	P97.5	28.28%	26.97%	29.69%	31.01%	28.97%	37.66%	36.10%	39.40%	41.25%	38.58%
	P99.9	49.67%	44.45%	56.06%	64.68%	53.14%	66.15%	58.23%	76.40%	90.68%	71.60%
4-6 years	P90	18.72%	18.26%	19.30%	19.85%	19.01%	24.53%	23.86%	25.24%	25.95%	24.88%
	P97.5	27.46%	26.43%	28.61%	30.05%	28.05%	35.83%	34.53%	37.54%	39.26%	36.73%
	P99.9	48.01%	43.27%	55.00%	66.55%	52.06%	63.31%	56.94%	73.06%	84.79%	68.68%
7-10 years	P90	16.75%	16.22%	17.25%	17.74%	16.99%	19.44%	18.86%	19.97%	20.50%	19.69%
	P97.5	24.48%	23.44%	25.63%	26.77%	25.06%	28.46%	27.38%	29.71%	31.11%	29.09%
	P99.9	42.61%	38.21%	49.40%	57.29%	46.11%	50.42%	44.37%	58.64%	67.36%	54.66%
11-13 years	P90	11.77%	11.47%	12.11%	12.47%	11.94%	14.61%	14.16%	15.03%	15.43%	14.81%
	P97.5	17.17%	16.51%	18.01%	18.77%	17.60%	21.41%	20.60%	22.49%	23.57%	21.97%
	P99.9	30.24%	27.19%	34.76%	42.56%	32.92%	37.81%	33.84%	43.49%	51.16%	40.87%
14-17 years	P90	11.03%	10.75%	11.37%	11.69%	11.21%	12.05%	11.73%	12.41%	12.80%	12.23%
	P97.5	16.13%	15.48%	16.92%	17.74%	16.54%	17.68%	17.01%	18.56%	19.42%	18.14%
	P99.9	28.23%	24.93%	32.93%	39.00%	30.75%	31.07%	27.76%	36.30%	42.97%	33.97%
18-29 years	P90	10.69%	10.38%	11.03%	11.28%	10.86%	11.97%	11.67%	12.29%	12.75%	12.14%
	P97.5	15.68%	15.08%	16.38%	17.06%	16.03%	17.57%	16.88%	18.41%	19.44%	18.03%
	P99.9	27.18%	24.06%	31.34%	38.22%	29.58%	31.34%	27.78%	35.90%	41.91%	33.81%
30-44 years	P90	10.04%	9.78%	10.33%	10.66%	10.19%	11.44%	11.09%	11.80%	12.19%	11.63%
	P97.5	14.72%	14.03%	15.44%	16.14%	15.07%	16.86%	16.15%	17.60%	18.64%	17.24%
	P99.9	25.89%	22.53%	29.76%	36.64%	28.02%	30.05%	26.44%	34.22%	39.70%	32.23%
45-59 years	P90	9.81%	9.56%	10.09%	10.41%	9.95%	11.25%	10.94%	11.60%	11.98%	11.42%
	P97.5	14.38%	13.82%	15.07%	15.84%	14.73%	16.68%	16.00%	17.46%	18.32%	17.07%
	P99.9	25.30%	22.55%	29.28%	33.86%	27.46%	29.58%	26.94%	34.03%	40.69%	32.03%
60-69 years	P90	9.59%	9.36%	9.93%	10.25%	9.77%	11.16%	10.83%	11.50%	11.87%	11.33%
	P97.5	14.12%	13.55%	14.85%	15.46%	14.48%	16.54%	15.87%	17.30%	18.17%	16.94%
	P99.9	24.93%	22.59%	29.02%	33.88%	27.22%	29.33%	26.16%	34.60%	40.45%	32.16%
≥70 years	P90	8.94%	8.75%	9.21%	9.50%	9.08%	10.00%	9.70%	10.34%	10.66%	10.17%
	P97.5	13.09%	12.56%	13.74%	14.41%	13.42%	14.87%	14.18%	15.58%	16.34%	15.22%
	P99.9	22.80%	20.78%	26.47%	30.96%	24.84%	26.78%	23.53%	30.82%	36.01%	28.89%

Table S6. Contribution distribution of dietary risks faced by different population groups.

Component	2-3 years old				30-44 years old				60-69 years old			
	urban		rural		urban		rural		urban		rural	
	male	female	male	female	male	female	male	female	male	female	male	female
Metalaxyl-M	15.42%	17.47%	17.41%	17.81%	19.46%	17.72%	23.78%	22.33%	18.15%	16.78%	22.05%	21.84%
Azoxystrobin	84.58%	82.53%	82.59%	82.19%	80.54%	82.28%	76.22%	77.67%	81.85%	83.22%	77.95%	78.16%
Rice and products	6.66%	6.53%	7.18%	7.34%	8.06%	6.67%	8.63%	8.04%	6.47%	5.81%	8.06%	7.82%
Flour and products	1.64%	1.93%	2.18%	1.65%	2.53%	2.02%	2.73%	2.33%	2.25%	2.10%	2.62%	2.21%
Other cereals	0.20%	0.21%	0.20%	0.16%	0.12%	0.10%	0.25%	0.23%	0.17%	0.16%	0.30%	0.28%
Potatoes and products	0.26%	0.32%	0.68%	0.71%	0.42%	0.36%	0.74%	0.73%	0.35%	0.38%	0.69%	0.71%
Legumes and products	0.66%	0.62%	0.70%	0.65%	0.78%	0.67%	0.84%	0.73%	0.81%	0.75%	0.84%	0.85%
Dark vegetables	28.79%	26.64%	34.87%	33.01%	36.56%	34.71%	37.68%	38.56%	37.78%	37.73%	41.79%	43.05%
Light vegetables	24.15%	27.01%	26.30%	26.27%	29.10%	26.59%	34.85%	33.85%	27.04%	25.08%	33.23%	32.93%
Fruits	32.95%	31.51%	24.03%	26.51%	16.36%	23.64%	9.22%	11.10%	19.41%	22.28%	7.81%	7.96%
Vegetable oil	0.63%	0.78%	0.44%	0.40%	0.72%	0.62%	0.59%	0.50%	0.69%	0.70%	0.54%	0.52%
Soy sauce	4.05%	4.47%	3.41%	3.29%	5.35%	4.60%	4.46%	3.93%	5.03%	5.01%	4.10%	3.67%
